# Supplementary material for: Low viscosity of the Earth’s inner core
Source: Nat Commun. 2019 Jun 6;10:2483. doi: 10.1038/s41467-019-10346-2 (PMC6554349; doi:10.1038/s41467-019-10346-2)
Supplement: Supplementary file 1 — Supplementary Information [file 41467_2019_10346_MOESM1_ESM.pdf]

Supplementary Information

Low viscosity of the Earth Inner Core

Anatoly B. Belonoshko et al.

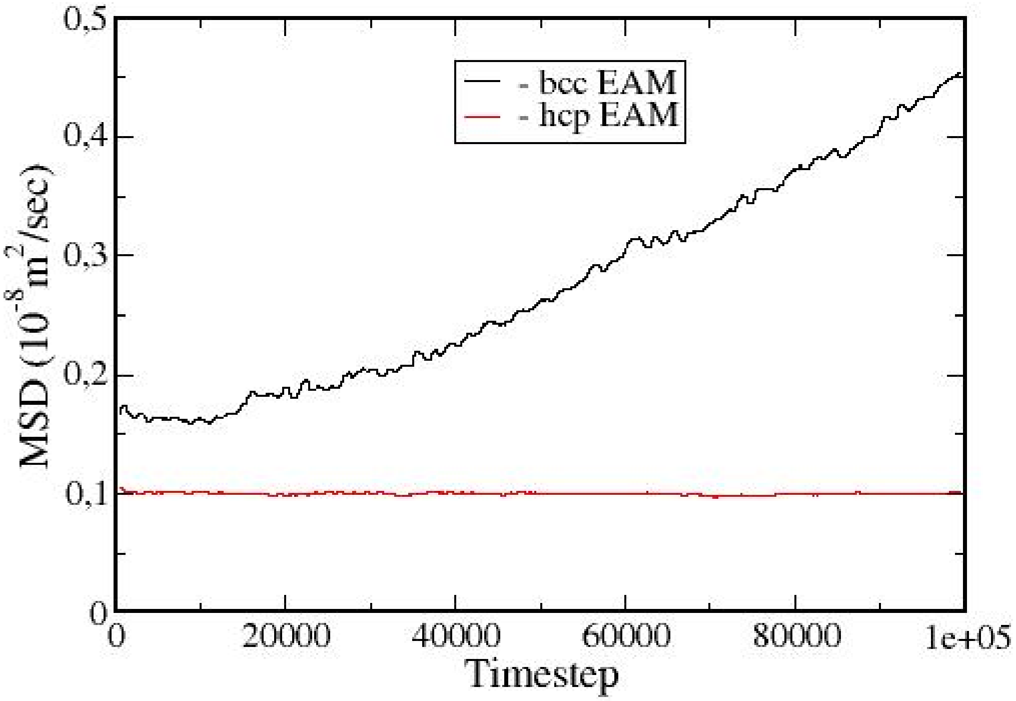

Supplementary Figure 1. Mean square displacement of atoms. Diffusion in the body-centered cubic and hexagonal close packed iron at pressure 360 GPa and 7000 K obtained by molecular dynamics using the embedded atom (EAM) model of interaction. While the diffusion in the bcc iron steadily increases there is no diffusion in the hcp iron. This is in accordance with ab initio calculations (Fig. 1). One timestep is equal to 0.001 picosecond. Note that the diffusion steady state in the body-centered cubic phase does not begin before 10,000 timesteps.

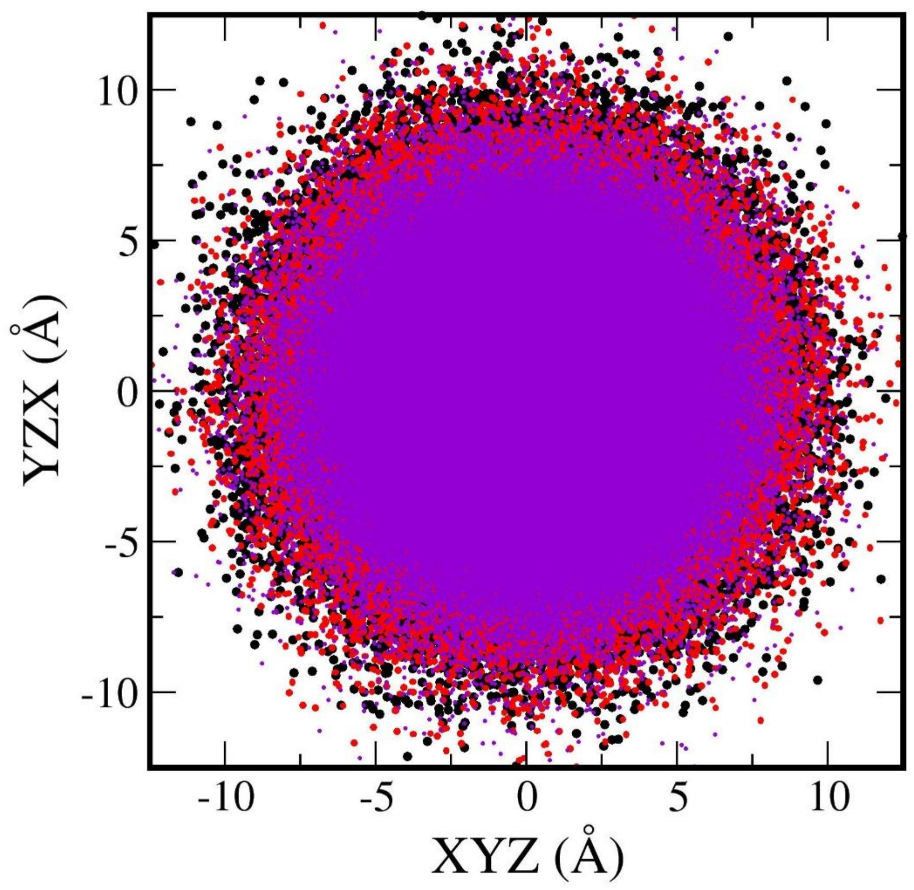

Supplementray Figure 2. Projections of displacements of atoms in liquid Ar. The axes for projections are given as triplets with 1-to-1 correspondence, that is XYZ-YZX correspond to X-Y, Y-Z, and Z-X projections. The displacements are shown by small circles with correspondingly black, red, and violet colors. The displacements of atoms in liquid Ar are similar to the displacements of Fe atoms in the body centered cubic iron.

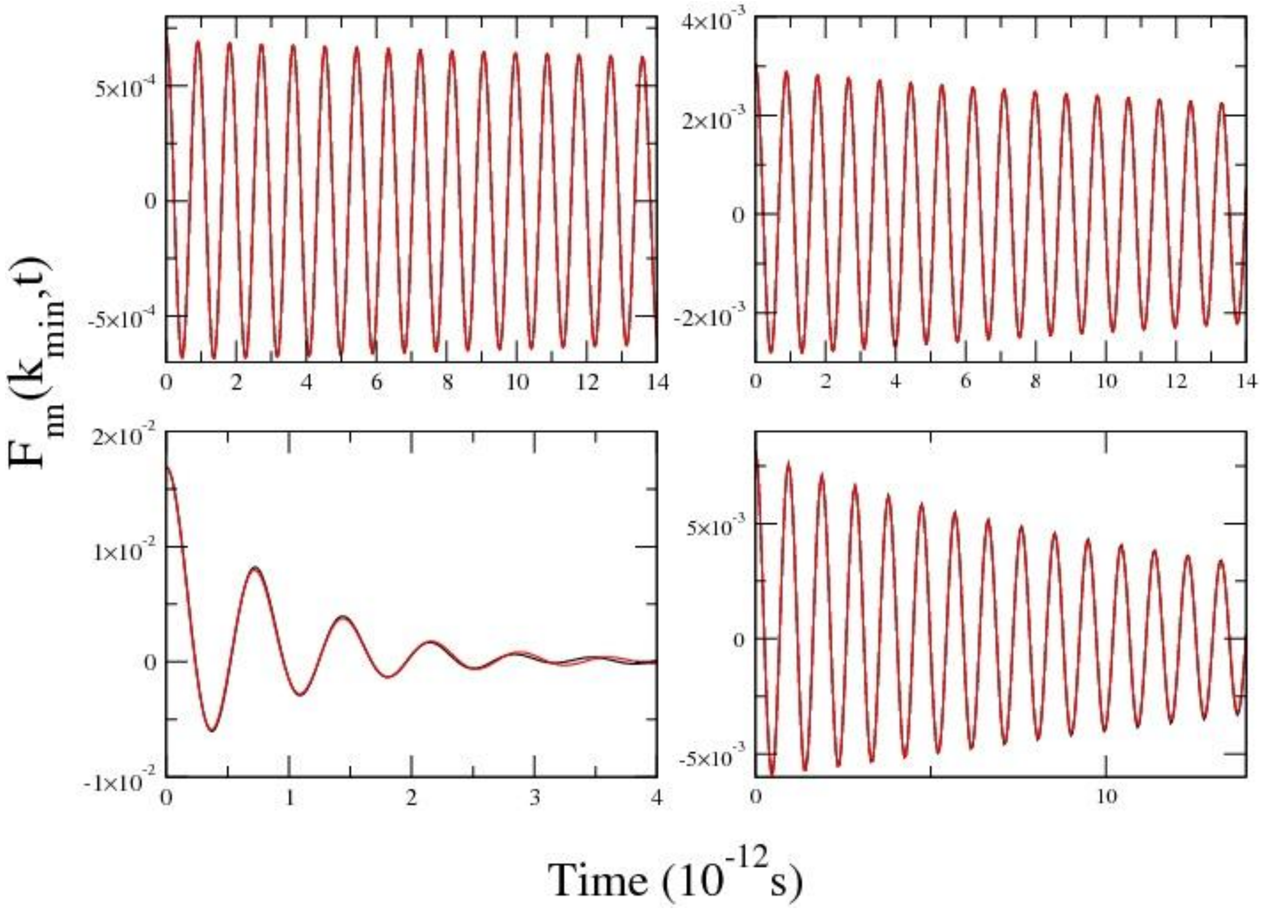

Supplementary Figure 3. Density-density time correlation functions in liquid and crystalline states. Red lines correspond to the simulation-derived time correlation functions, and black lines – to their fit by theoretical expressions (see Methods, equations 1 and 2). Upper left and right plots correspond to the Ar crystals described by the Lennard-Jones potential at 100 K and 300 K at the volume 23.0979 Å^3/atom, respectively. Left bottom frame corresponds to Ar liquid at 2500 K for the same volume, and the right bottom one - to bcc iron described by the EAM potential[32] at the volume corresponding to the pressure of 360 GPa at the temperature 7000 K. The wave number k_{min} is the smallest accessible in simulations and equals to $2\pi/L$, where L is the size of the cubic box.

Supplementary Table 1: Estimated from the fit sound damping coefficient *Γ*, speed of sound c_S and ratio of specific heats *γ*. Typical values of *γ* for dense Ar-liquids are 1.2-1.8.

system	Phase	T [K]	Γ [Å^2/ps]	c_S [Å/ps]	γ	D_T [A^2/ps]
Ar	liquid	2500	53.986	62.8	1.328	54.142

Supplementary Table 2. Estimated from the fit sound damping coefficient *Γ*, speed of sound c_S and weight coefficient of structural relaxation *A*, and structural relaxation time τ_σ.

System	phase	T [K]	Γ [Å^2/ps]	c_S [Å/ps]	A	τ_σ [ps]
Ar	cryst	100	0.395	49.92	0.0244	0.750
Ar	cryst	300	1.047	50.98	0.0449	0.6538
Fe	cryst	7000	20.360	124.90	0.1274	4.4263
